# Stapler-assisted extracorporeal revision for non-reducible prolapse of the efferent limb of a loop colostomy in advanced pelvic malignancy

**DOI:** 10.1093/jscr/rjag568

**Published:** 2026-07-10

**Authors:** Grace El Nabbout, Zein Maher Daher, Kelly J Manahan, John P Geisler

**Affiliations:** Trinity School of Medicine, PO Box 885, Ratho Mill, Kingstown, Saint Vincent and the Grenadines, West Indies; Trinity School of Medicine, PO Box 885, Ratho Mill, Kingstown, Saint Vincent and the Grenadines, West Indies; Trinity School of Medicine, PO Box 885, Ratho Mill, Kingstown, Saint Vincent and the Grenadines, West Indies; Trinity School of Medicine, PO Box 885, Ratho Mill, Kingstown, Saint Vincent and the Grenadines, West Indies

**Keywords:** stomal prolapse, loop colostomy, extracorporeal repair, stapler-assisted surgery, palliative surgery, minimally invasive technique, stoma complications

## Abstract

Ostomy prolapse can occur with any type of ostomy. Although it may be only a cosmetic concern in some patients, it can also lead to significant morbidity, including strangulation and bowel obstruction. We describe the case of a 47-year-old female with recurrent mucinous ovarian carcinoma, ~65 months after her initial diagnosis. She presented with large bowel obstruction caused by a 17 cm pelvic recurrence in the setting of platinum-resistant disease after her third course of chemotherapy. A diverting loop colostomy was performed. Three months later, she developed a large prolapse of the efferent limb, preventing proper ostomy appliance placement and significantly affecting quality of life. Conservative reduction attempts were unsuccessful. After informed consent, the patient underwent extracorporeal revision using gastrointestinal staplers. This minimally invasive approach avoided a major abdominal operation and allowed faster postoperative recovery, an important consideration given her advanced disease and palliative clinical setting.

## Introduction

Diverting ostomies, including colostomies and ileostomies, are commonly created for bowel obstruction, malignancy, and other gastrointestinal conditions. More than 100 000 new stomas are created annually in the United States [1]. Loop colostomies are frequently used in patients with unresectable malignancy or advanced large bowel obstruction to provide decompression and symptom relief [2, 3].

Despite their therapeutic benefits, ostomy-related complications remain common, affecting up to 70% of patients [3, 4]. Stomal prolapse occurs when the bowel telescopes through the stoma and is more frequently seen in loop ostomies, particularly involving the distal efferent limb [3, 5]. As prolapse progresses, patients may develop bleeding, ulceration, obstruction, incarceration, and difficulty with appliance fitting [3, 5].

Traditional management often involves laparotomy with bowel resection or stoma relocation; however, these procedures may carry substantial morbidity in medically frail or palliative patients [4, 6]. Extracorporeal stapler-assisted revision has emerged as a less invasive alternative associated with shorter operative times and faster recovery [7–10]. We present a case of successful stapler-assisted extracorporeal revision for a non-reducible prolapse of the efferent limb of a loop colostomy in a patient with advanced pelvic malignancy.

## Case

A 47-year-old woman with recurrent platinum-resistant mucinous ovarian carcinoma presented with large bowel obstruction secondary to a 17 cm pelvic recurrence ~65 months after her initial diagnosis and five months after her most recent platinum-based chemotherapy. Due to advanced disease, nutritional frailty, and palliative treatment goals, a diverting loop colostomy was performed to decompress the colon and relieve the obstruction. She was discharged 2 days later with adequate bowel function and was seen 1 week postoperatively with a functioning ostomy and no complications.

At 3-month follow-up, she was noted to have a small reducible prolapse of the efferent limb. At that time, she declined further oncologic therapy and hospice consultation.

Less than 1 month later, she presented to the emergency department with a massive non-reducible prolapse that prevented application of the ostomy appliance. Bedside reduction attempts by both the emergency department and gynecologic oncology teams were unsuccessful.

Management options were discussed in detail. Although the patient declined additional cancer-directed therapy, restoration of ostomy function remained important for comfort and quality of life. Due to the risks associated with laparotomy, including prolonged recovery and increased postoperative morbidity, an extracorporeal approach was selected.

**Figure 1:**
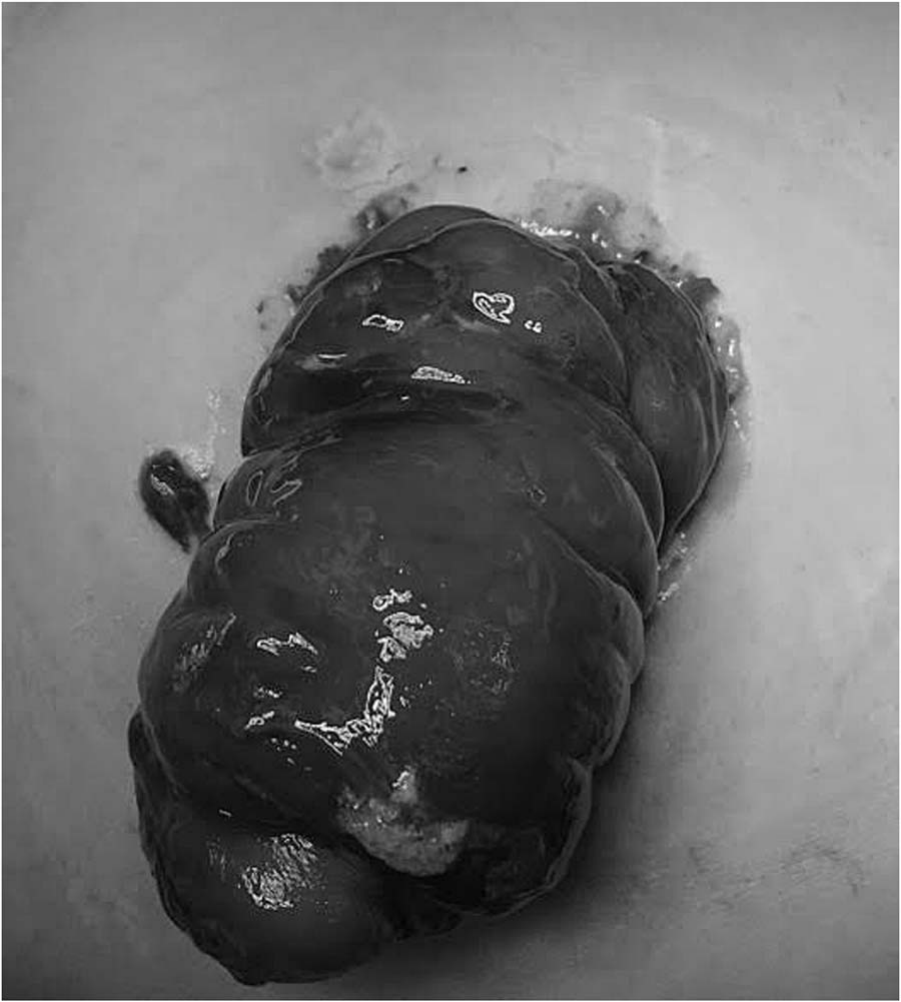
Partial reduction of the prolapsed efferent limb under general anesthesia prior to stapler-assisted revision.

After informed consent was obtained, the patient was taken to the operating room and underwent general anesthesia with bilateral paraspinal liposomal bupivacaine blocks. Under anesthesia, the prolapsed bowel was partially reduced (Fig. 1). However, the prolapsed segment continued to occupy most of the ostomy appliance, making long-term appliance use impractical.

**Figure 2:**
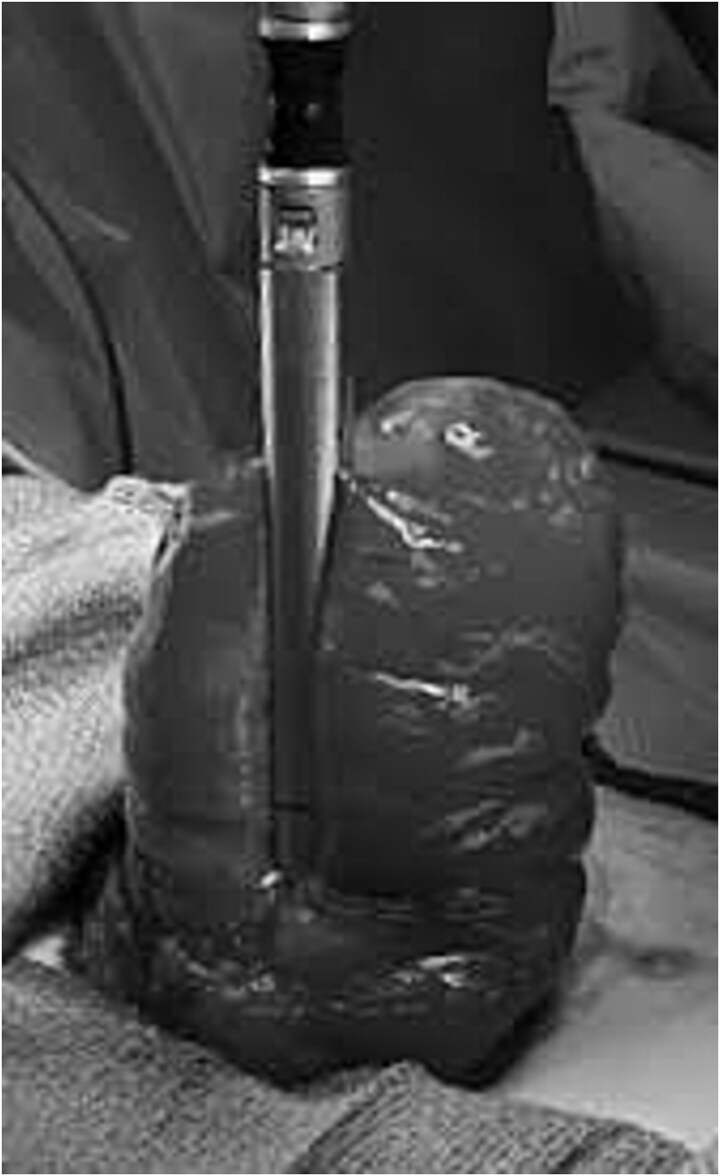
Initial placement of the 60 mm linear stapler across the prolapsed efferent limb at the 3 o’clock position.

**Figure 3:**
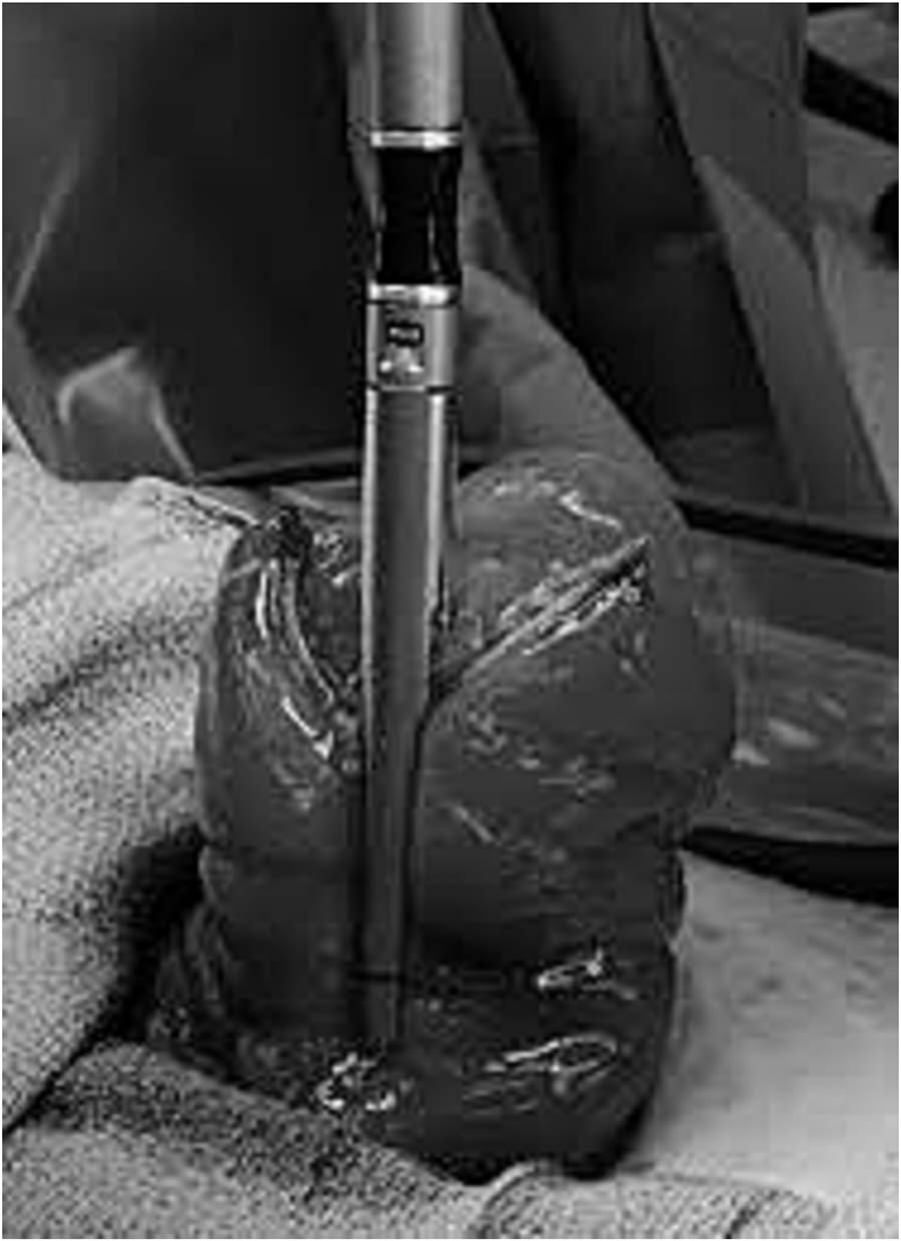
Sequential stapler division of the prolapsed bowel using extracorporeal technique.

**Figure 4:**
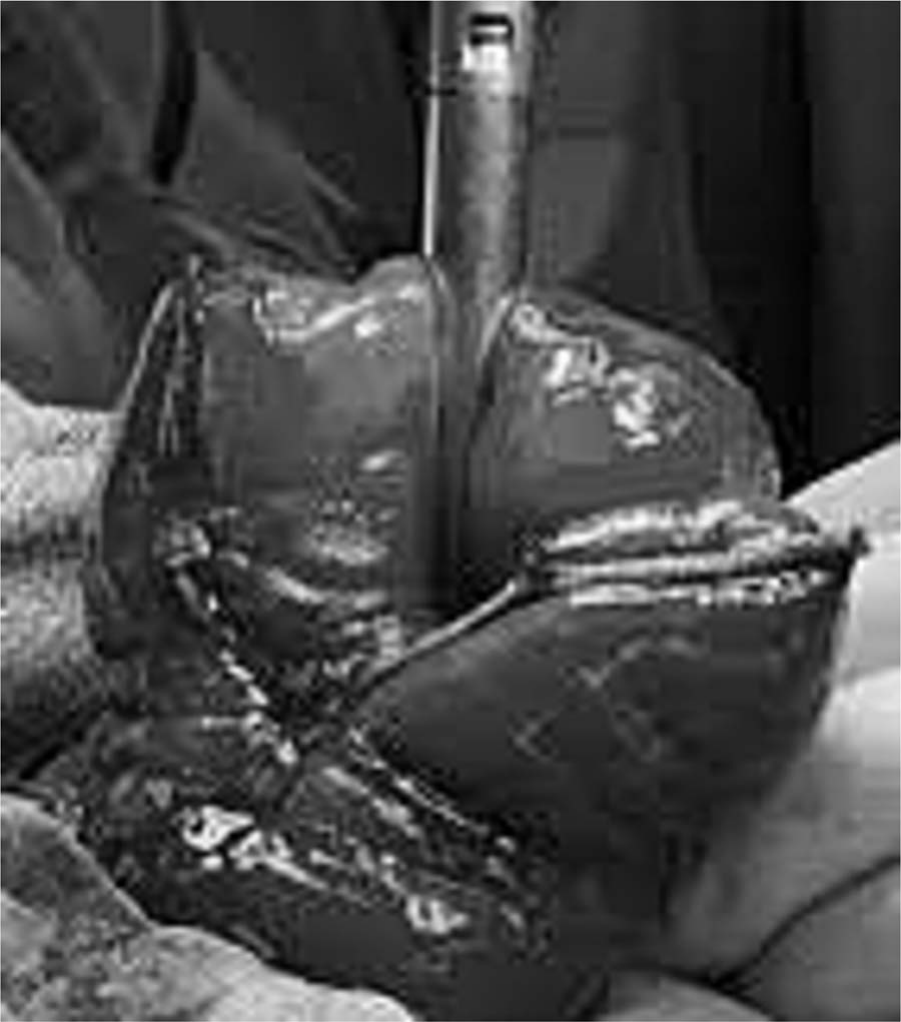
Continued stapler-assisted transection of the prolapsed segment with preservation of viable bowel.

**Figure 5:**
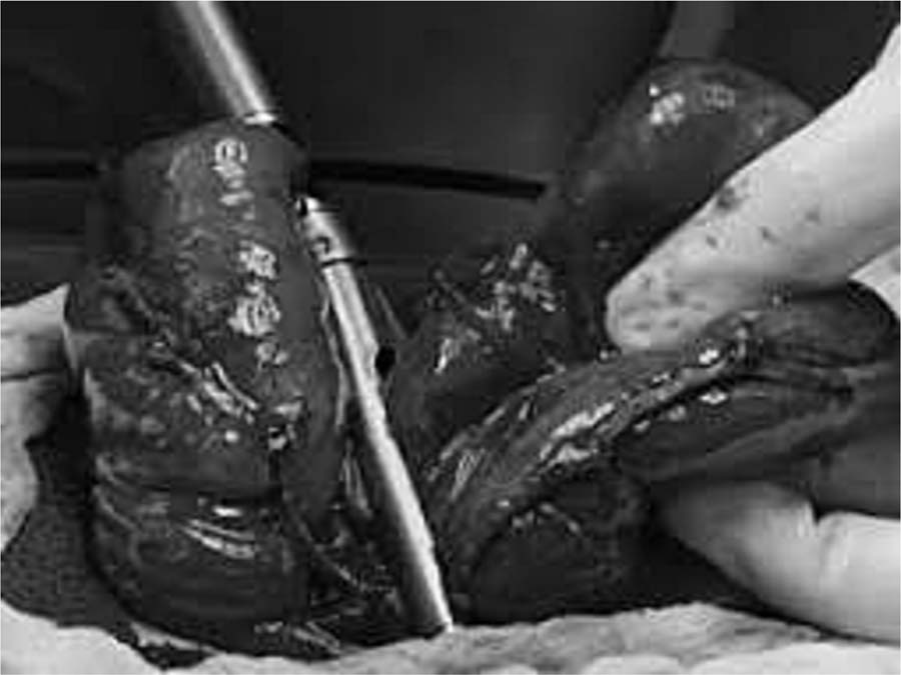
Completion of bowel division following successive stapler firings.

**Figure 6:**
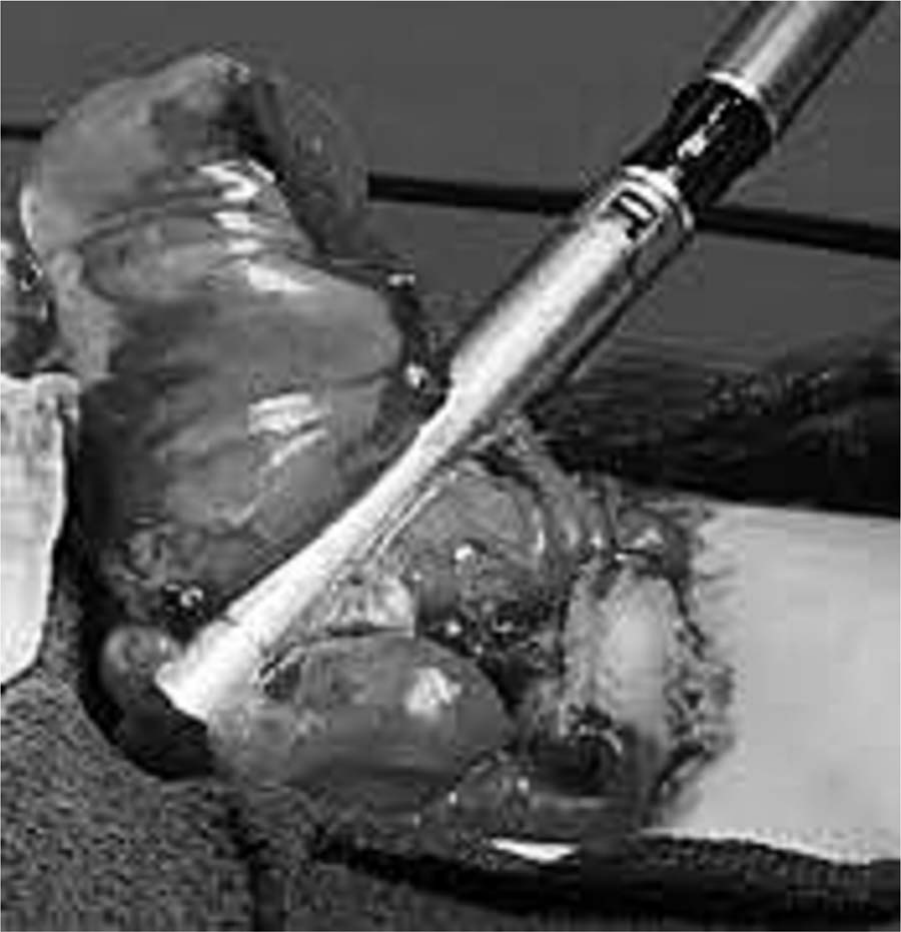
Final appearance of the revised stoma after extracorporeal stapler-assisted resection.

**Figure 7:**
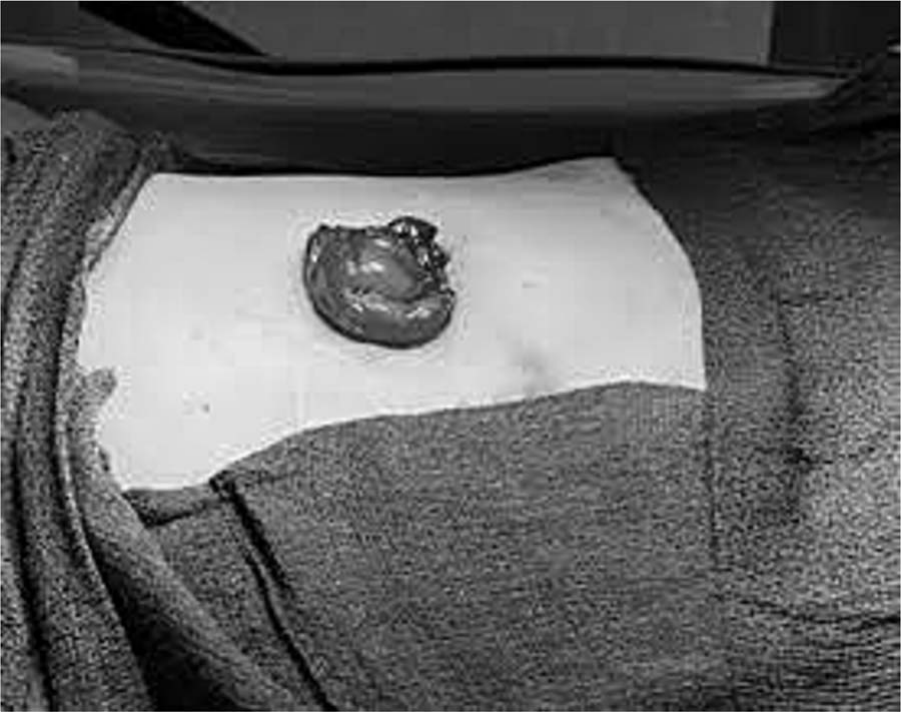
Functional ostomy following revision prior to appliance placement demonstrating successful reduction of the prolapse.

Because of tissue thickness, a 60 mm endoscopic linear stapler with 4.1 mm staples was used (Figs 1–4). Two sequential 60 mm staple loads were fired at the 3 o’clock and 9 o’clock positions to divide the prolapsed bowel. The remaining bowel was then transected with additional staple loads (Figs 5 and 6). Vascular supply was monitored intraoperatively with Doppler ultrasound. Figure 7 demonstrates the revised ostomy prior to appliance placement.

Postoperatively, pain was controlled with oral ibuprofen and acetaminophen alone, without narcotic requirements. The patient was discharged the following morning with a functioning colostomy and improved appliance fit. At one-week follow-up, she had no postoperative pain or recurrent prolapse. She died four months later from progression of her malignancy without recurrence of the prolapse.

## Discussion

Management of stomal prolapse in patients with advanced malignancy differs from management in patients undergoing curative treatment. In this population, treatment goals shift toward symptom relief, preservation of ostomy function, and reduction of physiologic stress rather than durable anatomic correction [3, 4]. In our patient with extensive pelvic disease and poor prognosis, extracorporeal stapler-assisted revision restored ostomy function and improved quality of life while avoiding a major abdominal operation.

Although laparotomy with stoma relocation or bowel resection remains a standard surgical option for stomal prolapse, its role may be limited in patients with progressive or terminal malignancy due to increased morbidity, prolonged recovery, and interruption of palliative care [3, 6, 4]. The MISSTO-WSES mapping review emphasized tailoring operative management according to prognosis, comorbidities, and overall treatment goals, recommending local or extracorporeal approaches when symptom control and functional improvement are prioritized [4]. In our patient with platinum-resistant ovarian carcinoma and extensive pelvic recurrence, these considerations supported avoidance of intraperitoneal surgery.

Stapler-assisted extracorporeal revision has previously been described as a minimally invasive alternative in high-risk surgical patients [7–10]. Tepetes *et al*. reported successful local stapler repair of prolapsed loop colostomy in a patient with metastatic rectal cancer, demonstrating minimal blood loss and uncomplicated postoperative recovery [7]. Hata *et al*. showed that stapler-assisted repair can be safely performed without entering the peritoneal cavity, while Monette *et al*. further demonstrated the efficiency and reliability of this technique in patients unsuitable for extensive surgery [8, 9]. Kosuge *et al*. additionally reported shorter operative times and low perioperative complication rates with stapler-assisted local repair compared with more extensive reconstructive procedures [10]. Lovisetto and Zonta similarly described effective extracorporeal treatment of loop colostomy prolapse in severely ill patients using an Altemeier-type peristomal resection [11].

This case demonstrates that extracorporeal stapler-assisted revision remains feasible even in patients with advanced pelvic malignancy and extensive local recurrence. By avoiding laparotomy, this approach minimized physiologic burden and shortened postoperative recovery while restoring ostomy function. Although limited by its single-case nature, this report supports extracorporeal stapler-assisted revision as a practical, patient-centered option for symptom management and quality-of-life improvement in palliative surgical care.

## Conclusion

Stapler-assisted extracorporeal revision offers a safe, minimally invasive option for managing non-reducible stomal prolapse in patients with advanced malignancy. By avoiding laparotomy, this approach reduces physiologic burden and supports continued palliative care, while effectively restoring ostomy function and improving the quality of life.

## References

[1] Wu JS. Intestinal stomas: historical overview. In: Fazio VW, Church JM, Wu JS (eds), *Atlas of Intestinal Stomas*. New York, NY: Springer, 2012, 1–37. 10.1007/978-0-387-78851-7_1

[2] Garofalo T . Colostomy: types, indications, formation, and reversal. In: Fazio VW, Church JM, Wu JS (eds), *Atlas of Intestinal Stomas.* Boston, MA: Springer, 2011, 127–145. 10.1007/978-0-387-78851-7_11

[3] Murken D, Bleier J. Ostomy-related complications. Clin Colon Rectal Surg 2019;32:176–82. 10.1055/s-0038-1676995 31061647 PMC6494607

[4] Parini D, Bondurri A, Ferrara F et al. Surgical management of ostomy complications: a MISSTO–WSES mapping review. World J Emerg Surg 2023;18:48. 10.1186/s13017-023-00516-5 PMC10563348 37817218

[5] Maeda K . Prolapse of intestinal stoma. Ann Coloproctol 2022;38:335–42. 10.3393/ac.2022.00465.0066 36353832 PMC9650348

[6] Garoufalia Z, Mavrantonis S, Emile SH et al. Surgical treatment of stomal prolapse: a systematic review and meta-analysis of the literature. Color Dis 2023;25:1128–34. 10.1111/codi.16548 36965087

[7] Tepetes K, Spyridakis M, Hatzitheofilou C. Local treatment of a loop colostomy prolapse with a linear stapler. Tech Coloproctol 2005;9:156–8. 10.1007/s10151-005-0217-2 16007355

[8] Hata F, Kitagawa S, Nishimori H et al. A novel, easy, and safe technique to repair a stoma prolapse using a surgical stapling device. Dig Surg 2005;22:306–10. 10.1159/000088626 16192729

[9] Monette MM, Harney RT, Morris MS et al. Local repair of stoma prolapse: case report of an in vivo application of linear stapler devices. Ann Med Surg 2016;11:32–5. 10.1016/j.amsu.2016.08.018 PMC5024141 27668078

[10] Kosuge M, Ohkuma M, Koyama M et al. Evaluation of the outcome of local surgery for stomal prolapse. J Clin Med 2021;10:5438. 10.3390/jcm10225438 34830719 PMC8622099

[11] Lovisetto F, Zonta S. Altemeier’s procedure for loop colostomy prolapse in severe patients: our experience. Clin Surg 2021;6:3079.

